# The genome of sheep ked (*Melophagus ovinus*) reveals potential mechanisms underlying reproduction and narrower ecological niches

**DOI:** 10.1186/s12864-023-09155-1

**Published:** 2023-01-30

**Authors:** Qingxun Zhang, Qingsong Zhou, Shuyi Han, Ying Li, Ye Wang, Hongxuan He

**Affiliations:** 1 National Research Center for Wildlife-Borne Diseases, Institute of Zoology, Chinese Academy of Sciences, Beijing, 100101 China; 2 Beijing Milu Ecological Research Center, Beijing, 100076 China; 3 Key Laboratory of Zoological Systematics and Evolution, Institute of Zoology, Chinese Academy of Sciences, Beijing, 100101 China; 4 State Key Laboratory of Plateau Ecology and Agriculture, Qinghai University, Xining, 810016 China

**Keywords:** *Melophagus ovinus*, Pathogen diversity, Genetic diversity, Whole-genome, Gene family evolution

## Abstract

**Background:**

*Melophagus ovinus* is considered to be of great veterinary health significance. However, little is known about the information on genetic mechanisms of the specific biological characteristics and novel methods for controlling *M. ovinus*.

**Results:**

In total, the de novo genome assembly of *M. ovinus* was 188.421 Mb in size (330 scaffolds, N50 Length: 10.666 Mb), with a mean GC content of 27.74%. A total of 13,372 protein-coding genes were functionally annotated. Phylogenetic analysis indicated that the diversification of *M. ovinus* and *Glossina fuscipes* took place 72.76 Mya within the Late Cretaceous. Gene family expansion and contraction analysis revealed that *M. ovinus* has 65 rapidly-evolving families (26 expansion and 39 contractions) mainly involved DNA metabolic activity, transposases activity, odorant receptor 59a/67d-like, IMD domain-containing protein, and cuticle protein, etc. The universal and tightly conserved list of milk protein orthologues has been assembled from the genome of *M. ovinus*. Contractions and losses of sensory receptors and vision-associated Rhodopsin genes were significant in *M. ovinus*, which indicate that the *M. ovinus* has narrower ecological niches.

**Conclusions:**

We sequenced, assembled, and annotated the whole genome sequence of *M. ovinus*, and launches into the preliminary genetic mechanisms analysis of the adaptive evolution characteristics of *M. ovinus*. These resources will provide insights to understand the biological underpinnings of this parasite and the disease control strategies.

**Supplementary Information:**

The online version contains supplementary material available at 10.1186/s12864-023-09155-1.

## Background

The family Hippoboscidae (Diptera, Hippoboscoidea) is closely associated with mammals and birds in its ecological habits [1] which contain 213 described species until now. Among them, *Melophagus ovinus*, commonly known as sheep ked, is a serious hematophagous pest with a limited host range, feeding mainly on sheep and goats and occasionally livestock (rabbits and dogs), and wild animals (Tibetan antelope, European bison, and red foxes) [2]. The presence of *M. ovinus* has been reported in most temperate sheep-rearing areas in Europe, Asia, North America, Africa, and Oceania [3].

*M. ovinus* is the most studied species of the family *Hippoboscidae* due to its veterinary importance and significant economic effects. *M. ovinus* infection results in skin damage, anemia, inflammation, wool loss, and subsequent secondary bacterial infections and cutaneous myiasis [2, 4]. *M. ovinus* has been reported to serve as potential vectors of zoonotic pathogens including Bluetongue virus, *Trypanosoma* spp., *Bartonella* spp., *Anaplasma* spp., *Borrelia burgdorferi*, Border disease virus, *Rickettsia* spp., *Theileria* spp [5–10]. Recent studies on the species have mainly focused on the diversity of pathogenic organisms carried by *M. ovinus* [3] and gut microbiota and obligate endosymbiont [11, 12]. Host-symbiont interactions between the keds and its obligate endosymbiont seem to influence their vector role [1].

Due to the highly specialized blood-feeding style, *M. ovinus* has developed a large number of specific adaptions in morphology and physiology. The life cycle of *M. ovinus* comprises three definite stages: larva, pupa, and wingless adult. Compared with the other hippoboscids such as *Glossina morsitans*, all life stages of sheep ked occur on the host, being strictly host dependent. Frequent direct contact between host animals makes it relatively easy for the offspring to transfer to a wide variety of hosts. The unique host ecology may explain the complete loss of wings in *M. ovinus* [13]. The reproductive biology of adenotrophic viviparity is unique to the superfamily Hippoboscoidea. Expansion and adaptation of the female accessory gland and the uterus provide the nutrient synthesis and delivery and the habitat for developing larvae [14, 15]. *M. ovinus* also adopt such reproductive mode and produce a single 3rd instar larva which immediately molded into pupae. During the whole lifecycle, the female produces only 12–15 progeny. The remarkable reproduction pattern of *M. ovinus* is considered to be the result of adaptive evolution, making their offspring with higher survival but lower reproductive capacity than other dipteran insects. Similarly, *M. ovinus* have highly specialized piercing-sucking mouthparts that are used for feeding on host blood and both females and males are strictly blood-feeding [2, 13]. Despite this, it remains unclear which genetic mechanisms form the basis for these long-term adaptive evolution characteristics.

Although the species of Hippoboscoidea are of veterinary and ecological importance, fewer genomic resources have been annotated [15, 16] seriously hindering investigation of the specific biological mechanisms (pathogen vectors and behavior characteristics) from the perspectives at genomic levels. Presently, genome-wide datasets are available for six medical and veterinary important ectoparasites from Glossinidae, which provide insight into the evolutionary biology and vector control strategies [16]. However, little is known about the information on genomes representing most families of Hippoboscidae, including *M. ovinus*. Here, we assembled a draft genome for *M. ovinus* and conducted comparative genome analyses with Glossinidae and other dipteran insects. This work gives first insights into the molecular biology of this hematophagous ectoparasite and provides a new resource for future studies of *M. ovinus*, and comparative genomic and genetic investigations of Hippoboscoidea.

## Results and discussion

### Genome assembly and annotation

In total, we generated 143.85 Gb of subreads with high sequencing depth (757.09 ×) on the PacBio Sequel System. A total of 21.32 Gb and 8.42 Gb clean data generated from the Illumina HiSeq X Ten platform for genome and transcriptome sequencing were used to correct the primary PacBio assembly. The genome size estimated by genomoscope 2.0 was 183.278 Mb (Figure S2). The de novo genome assembly of *M. ovinus* was 188.421 Mb in size (330 scaffolds N50 Length: 10.666 Mb), with a mean GC content of 27.74% and a heterozygosity of 0.456% on the basis of k-mer statistics (Table S1). Assembly completeness of *M. ovinus* (94.5%) was determined by Benchmarking Universal Single-Copy Orthologs (BUSCO) analysis, which demonstrated a highly accurate assembly (Table S2). The *M. ovinus* genome is slightly larger than that of *Drosophila melanogaster* (180 Mb) and much smaller than that of *Glossina* species (315–380 Mb) and *Musca domestica* (691 Mb) [16–18]. Genomic GC content is significantly correlated with biological features of genome organization [19]. The lower GC content in *M. ovinus* (27.74%) relative to other *Glossina* species (the GC content ranges from 27 to 35%) [16] could result in lower transcriptional activity of heterochromatic DNA. Furthermore, we assembled a complete mitochondrial (mtDNA) genome sequence of *M. ovinus*, which showed 99.93% and 99.04% sequences similarity with the previously published sequences (Genebank Accession NO.MH024396 and NC_037368).

A total of 13,372 proteins were identified by the algorithm MAKER with an average number of 3.79 exons and 11.06 introns per gene. Mean length of gene, exon, and intron were 4,477.1, 525.8, and 198.5 bp, respectively (Table 1), smaller than the findings for the genomes of *Glossina* species. We identified 9,505 genes with homology to proteins in the InterPro database, among which 6,320 were assigned to GO classifications, and 663, 525, and 2,609 genes could be annotated in the KEGG, MetaCyc, and Reactome pathway databases, respectively.

**Table 1:** Results of *de novo* genome of *M. ovinus*

	**Parameter**	**Number**
Scaffolds	N50 Length (Mb)	10.666
	No. Scaffolds	330
	Longest Scaffold (Mb)	24.548
Genome	Total Length (Mb)	188.421
	GC (%)	27.74
	Gene number	13,372
	Genomic completeness (%)	94.5
	Heterozygosity (%)	0.456
Gene annotation	Coding gene	13,372
	Average gene length (bp)	4,477.1
	Average cds length (bp)	447.5
	Exon number	50,680
	Average exon length (bp)	525.8
	Intron number	147,850
	Average intron length (bp)	198.543
	Repeat content (%)	27.08
ncRNA	rRNA	330
	miRNA	57
	snRNA	28
	ribozyme	3
	tRNA	153
	others	96

The estimated percentage of masked repeats this draft genome is 27.08%, in contrast to 34.95–39.99% of the *Glossina* species [16]. The most-abundant repeat types were unclassified repeats (14.28%), DNA/simple repeat (10.53%), DNA/low complexity (1.70%), LINE families (0.44%), LTR retro-elements (0.10%), and DNA/TcMar-Mariner (0.02%) (Table S3). In addition, 330 rRNAs, 57 miRNAs, 28 snRNAs, 3 ribozymes, 153 tRNAs, and 96 other ncRNAs were identified in the assembled genome (Tables 1; S4).

### Gene family analysis and evolution

Finally, 17,597 gene families were identified using OrthoFinder, covering 245,501 genes. Among these, 5,185 gene families were shared by all species, 325 were single-copy orthogroups, and 187 gene families containing 571 genes were unique to *M. ovinus* (Figure S3; Table S5). We then identified 325 shared single-copy orthologues to construct phylogenetic trees. The phylogeny analysis indicated that nine fly species were found to be clustered together into a large branch and strongly supported with high values (bootstrap = 100). *M. ovinus* was most closely related to *Glossina fuscipes* (*G. fuscipes*), and that these two species in turn formed a clade, supporting the sister relationship previously published between Glossinidae and Hippoboscidae [13]. The estimated divergence time between *D. melanogaster* and other eight fly species was 129.10 Mya, which was consistent with the result of recent studies [20]. To the Hippoboscoidea superfamily, the estimated divergence time was 89.09 Mya. Remarkably, the diversification of *M. ovinus* and *G. fuscipes* took place 72.76 Mya within the Late Cretaceous. These results suggested that there were considerable genetic distances between such species.

**Fig. 1:**
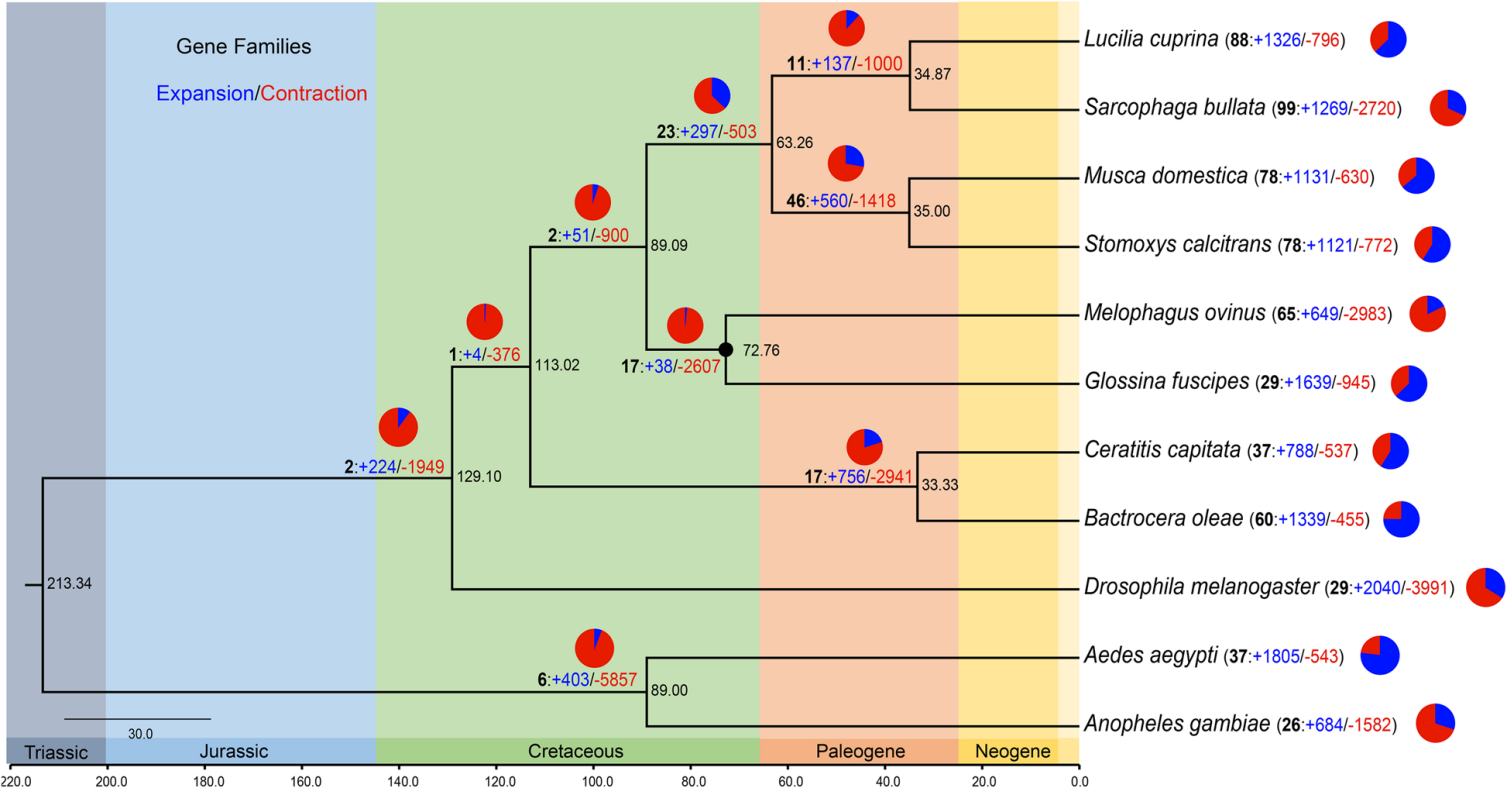
Comparative genomic analyses among *M. ovinus* and ten other species

Gene family expansion and contraction were analyzed using CAFE and gene birth rate was estimated at 0.00120 when accounting for duplications/gene/Mya. This approach revealed a total of 626 gene families for 11 species experienced significant expansion or contraction events (Fig. 1). Among them, *M. ovinus* has 65 rapidly-evolving families (26 expansion and 39 contractions). The corresponding genes identified from these gene families included 649 expanded genes and a greater degree of 2,983 contracted genes. The expanded families mainly involved DNA metabolic activity, transposases activity, DNA ligase activity, and DNA recombination (Figure S4 and Tables S6-7). In terms of contracted gene families in *M. ovinus*, these genes encoded odorant receptor 59a/67d-like, IMD domain-containing protein, cuticle protein, troponin, chloride-channel proteins, and fibrinogen C-terminal domain-containing protein, etc. (Table S7).

### Reproductive genetics of *M. ovinus*

In tsetse flies, nutrients for intrauterine larval development are synthesized by specialized glands and have been described as a novel milk-specific protein family. To date, thirteen milk proteins in have been characterized in tsetse flies including milk gland proteins 1–10 (*mgp*1-10), *transferrin*, *acid sphingomyelinase* 1, and Peptidoglycan Recognition Protein-LB (PGRP-LB) [16, 21]. A list of milk protein orthologues has been assembled from the genome of *M. ovinus*, which indicates that milk proteins are universal and tightly conserved in the Hippoboscoidea superfamily.

**Fig. 2:**
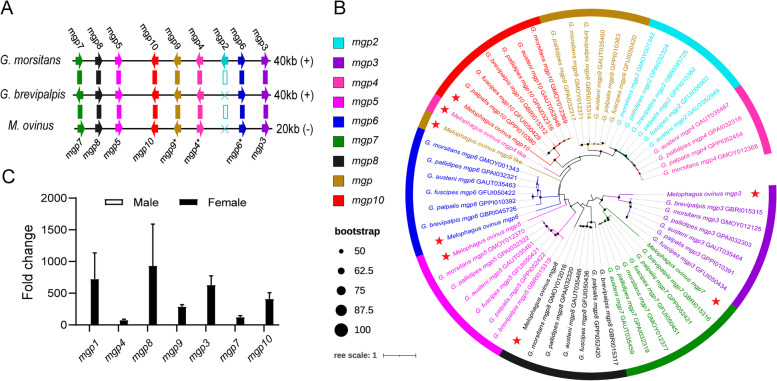
Overview of the conservation of *M. ovinus* milk protein genes and their expression patterns. **A** Syntenic analysis of gene structure/conservation in the *mgp*2-10 genetic locus. **B** Phylogenetic analysis of orthologs from the *mgp2-10* gene family. **C** Sex-specific expression of *M. ovinus* milk proteins

Search for orthologous sequences to *mgp*2-10 revealed that *M. ovinus* lacks an orthologous sequence for the *mgp*2 gene, while *G. brevipalpis* lacks the same *mgp*2 gene. The *mgp*2-10 gene family of *M. ovinus* shows a conserved sequential distribution pattern, while localizes to a 20 kb microsyntenic region with gene opposite-direction (Fig. 2A). Phylogenetic analysis showed that these genes can be divided into two distinct groups, one consisting of *mgp*3, 7, the other of *mgp*2, 4, 5, 6, 8, 9, 10. Among them, the *mgp*3, 7, 8, 5, 10 genes of *M. ovinus* clustered together with the corresponding genes of tsetse flies, but not the *mgp*6*, 4*, 9* genes, which probably indicated different evolutionary features between *M. ovinus* and tsetse flies (Fig. 2B). Recent studies showed the *mgp*2-10 gene family was defined as a highly divergent protein family and lack conserved functional protein domains [16, 21]. Evolutionarily related sequences of *M. ovinus* reveals a high degree of genetic diversity of milk genes, meanwhile, much more research in other viviparous genera will be helping shed light on their potential origin. Similar to *Glossina* species, comparative expression analysis of milk genes in male and female specimens showed that female-specific expression profiles (Fig. 2C). Interference with at least two milk proteins has substantial influence over tsetse fecundity and larval development [21–23]. In view of important role of these milk proteins in reproduction, functional characterization is warranted, as they might provide novel targets for the control of viviparous insects.

In addition, *M. ovinus* has a single yolk protein gene that is orthologous to *Glossina* and *M. domestica yp2* (Figure S5). In comparison with other fly species such as *M. domestica* (7 yolk proteins) and *D. melanogaster* (3 yolk proteins) [24], *M. ovinus* and *Glossina* have a marked reduction in yolk genes associated with their reduction in reproductive capacity.

### Immune characteristics of *M. ovinus*

**Fig. 3:**
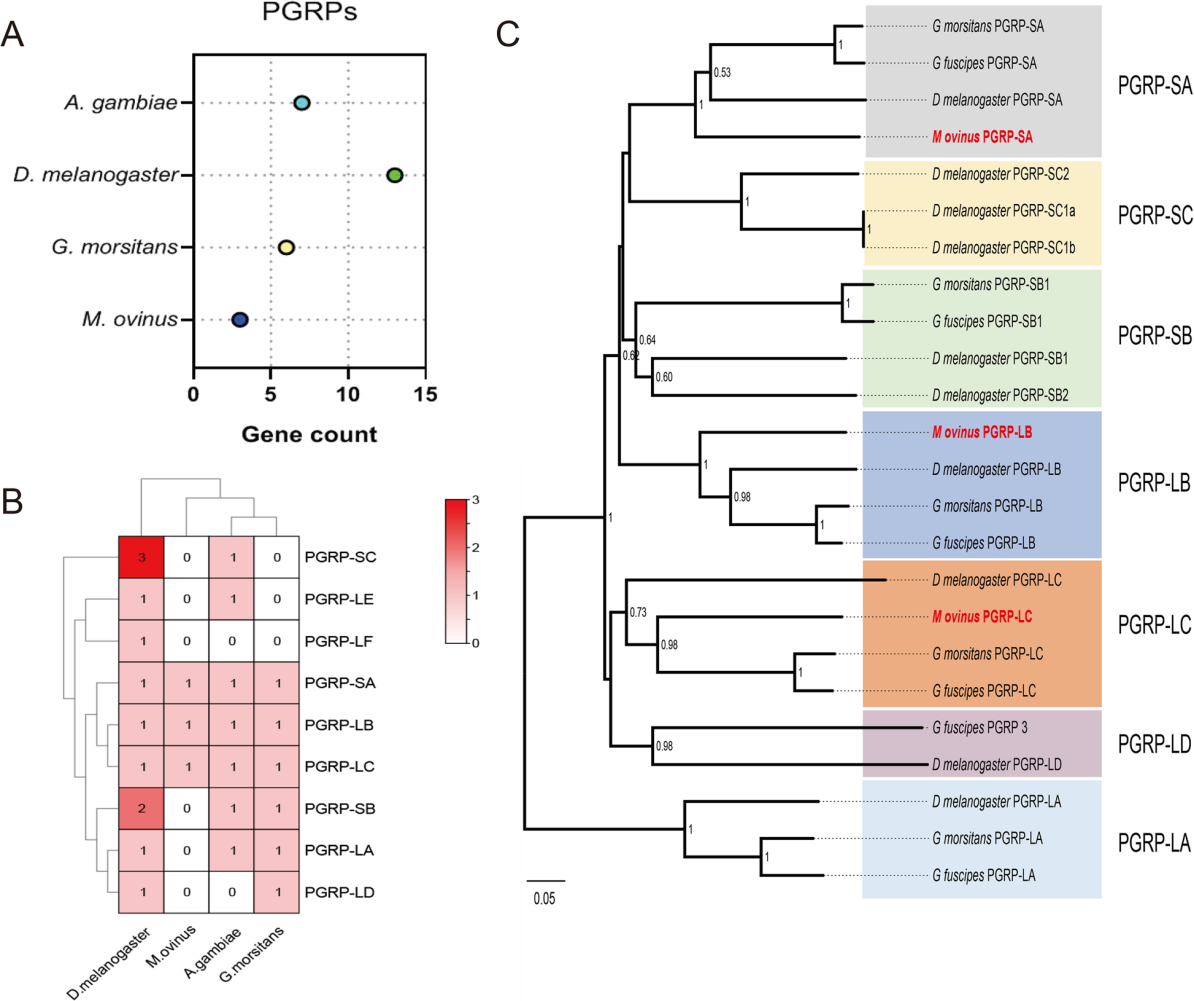
Distribution pattern and phylogenetic analysis of PGRPs orthologs. Total number (**A**), types and distribution (**B**) of the PGRPs. **C** Phylogenetic analysis of PGRPs orthologs

Peptidoglycan recognition proteins (PGRPs) are important pathogen-associated molecular patterns (PAMPs) involved in pathogen molecular recognition in insects and played an important role in natural antibacterial immunity and regulation of intestinal homeostasis [25, 26]. In *M. ovinus*, only three PGRP genes were identified, two in the long subfamily (PGRP-LB and -LC, and -SA) and one in the short subfamily (PGRP-SA), and *Glossina* has 6 PGRPs (4 long PGRP-L genes, 2 Short PGRP-S), whereas *D. melanogaster* has 13 PGRPs (6 long PGRP-L genes, 7 short PGRP-S), and *A. gambiae* has 7 PGRPs (4 long PGRP-L genes, 3 Short PGRP-S) (Fig. 3A-B). Phylogenetic analysis indicated that the PGRP-LB, PGRP-LC and PGRP-SA of the *M. ovinus* were clustered with the corresponding genes of the *Glossina* and *D. melanogaster*, respectively. Among them, PGRP-LB and PGRP-SA of sheep louse were phylogenically closer to *D. melanogaster*, while PGRP-LC is closer to *Glossina* (Fig. 3C). This indicates that PGRPs genes may have different ways in evolution.

The reduced PGRP repertoire of *M. ovinus* and *Glossina* might reflect its blood-specific diet, which likely exposes its gut to fewer microbes than other insects. While, the loss of PGRPs such as PGRP-SC1 and PGRP-LE might indicate that the immune system has evolved to protect the symbiosis of *M. ovinus*. This reduced immune capacity was also observed in tsetse flies and aphids which harbor obligate symbionts [15, 27]. PGRP-LB is a maternally transmitted immune milk protein and plays a pivotal role in tsetse flies by protecting the obligate symbiont *Wigglesworthia* [28]. In *M. ovinus*, the PGRP-LB sequence is highly homologous with *Glossina* PGRP-LB and may have functional relationship. Although phylogenetically distant with the mutualistic *Wigglesworthia* in *Glossina*, the obligate symbiont *Arsenophonus* of *M. ovinus* displays remarkably similar traits such as transmission via the milk glands and vital function in development and fecundity [29]. Hence, the PGRP-LB-*Arsenophonus* axis in *M. ovinus* might an analogy to the PGRP-LB-*Wigglesworthia* axis in tsetse flies.

### Contractions and losses of sensory receptors in *M. ovinus*

**Fig. 4:**
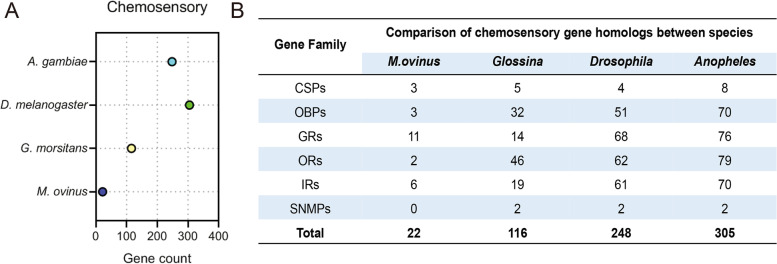
Comparison of sensory receptors gene homologs between species. Total number (**A**), types and distribution (**B**) of the sensory receptors

Insects with diversified ecological and biochemical niches impact the size of sensory receptor families with evidence for generalists insects (broad niches) having a larger number of genes compared with specialist (narrower niches) [24, 30]. These gene families encode chemosensory proteins (CSPs), gustatory receptors (GRs), odorant binding proteins (OBPs), odorant receptors (ORs), ionotropic receptors (IRs), and sensory neuron membrane proteins (SNMPs) [31, 32]. Detailed annotation of *M. ovinus* reveals that they have fewer sensory receptors (22) relative to *Glossina* (116), *D. melanogaster* (248), and *A. gambiae* (305) (Fig. 4). The chemoreceptor of the blood-sucking insect (*M. ovinus* and *Glossina*) is lower in number than the polyphagous insect (*D. melanogaster* and *A. gambiae*). Through the comparative analysis of homologous genes, it was found that the ORs and IRs of the *M. ovinus* were significantly lower than other insects. In addition, GRs associated with sweet tastes, present in *D. melanogaster* and *A. gambiae*, are missing in *M. ovinus* and *Glossina*. These genetic differences are consistent with the combination of a restricted diet of vertebrate blood and their narrow host range [33]. Compared with *Glossina*, a list of ORs and IRs of the *M. ovinus* has been lost over evolutionary history. Meanwhile, results from rapidly-evolving families showed that odorant receptor 59a-like and odorant receptor 67d-like were contracted. These results might explain the mechanism of the *M. ovinus* obligately parasitize sheep, while *Glossina* have a variety of animal hosts and a diverse living environment.

### Vision-associated Rhodopsin genes in *M. ovinus*

**Fig. 5:**
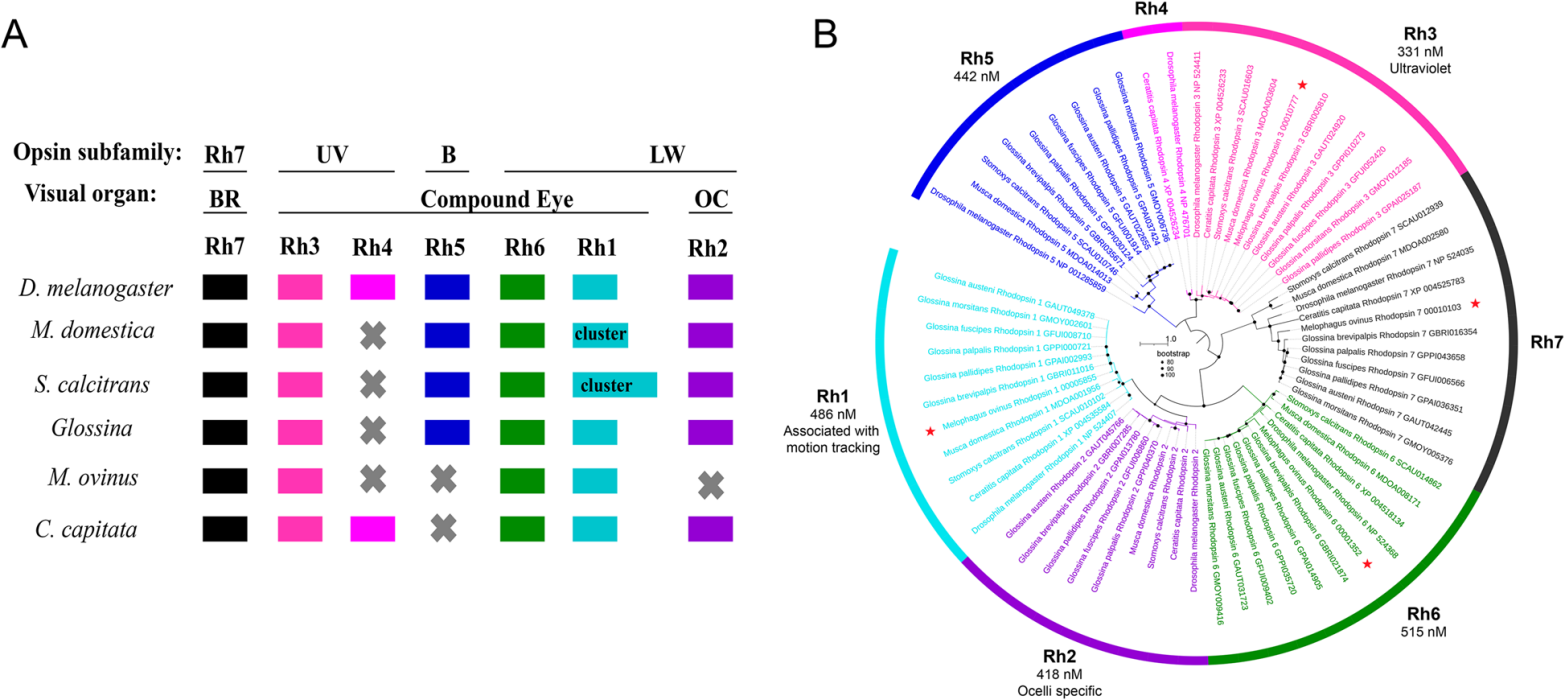
Distribution (**A**) and phylogenetic analysis (**B**) of orthologues from the Rhodopsin gene family

Vision-associated Rhodopsin genes play a critical role in host identification and pursuit and mate-seeking by insects [34, 35]. These color sensing Rhodopsin genes are identified as critical factors in the optimization and development of trap/target technologies [36, 37]. The visual system of *Glossina* is very similar to that of other *Brachycera* species, especially detecting colors in the blue wavelength ranges [15]. The search for vision-associated genes across the *M. ovinus* reveals orthologs to those described in the *Glossina* species. Unlike *Glossina*, which has six Rhodopsin genes (*Rh1*, *2*, *3*, *5*,* 6*, and *7*), *M. ovinus* has four Rhodopsin genes (*Rh1*, *3*, *6*, and *7*). Morphology and function of the compound eye retina is relative conserved throughout the *Brachycera* [15]. Five opsin genes are differentially expressed in the photoreceptors of the *D. melanogaster* compound eye, four of five in *Glossina*, *M. domestica*, and *S. calcitrans*, three of five in *M. ovinus* (Fig. 5A). The *Rh1* subfamily is deployed in motion vision [16] and shows greater sequence conservation between *M. ovinus* and *Glossina*. The loss of one of the two dipteran ultraviolet (UV)-sensitive Rhodopsins (*Rh4*) is found in *M. ovinus, Glossina*, *M. domestica*, and *S. calcitrans*. The blue sensitive opsin *Rh5*, which is expressed in color-discriminating inner photoreceptors [16], have been missed in *M. ovinus* and *C. capitata*. The common ocellus-specific opsin *Rh2* in other insects is not detected because of *M. ovinus* has no ocellus. In addition, the *M. ovinus* genome also contains the ortholog of the Rh7, which is recently characterized UV-like and circadian photoreceptor [38, 39]. Phylogenetic analysis suggests the Rhodopsin gene family contracted in *M. ovinus* after their divergence within the family Hippoboscidae. Besides, the Rhodopsin genes of the *M. ovinus* were clustered together with the corresponding genes of the *Glossina* species (Fig. 5B), which were relatively conservative in evolution and more closely related to each other. These genetic differences could account for the attractivity of trap and targets to different insects.

## Conclusion

In summary, we report a high-quality draft genome of *M. ovinus* that enabled the molecular landscape of this hematophagous ectoparasite to be characterized for the first time. This 188.421 Mb genome, with a repeat content of 27.08%, encodes 13,372 protein-encoding genes. Conspicuous in the predicted proteome are milk-specific protein family, PGRPs, sensory receptors, and Rhodopsin gene family, some of which appear worthy of investigation as novel targets for vector control strategies. In addition, comparative genomics allowed a re-evaluation of the genetic relationships of *M. ovinus* and *Glossina*. In conclusion, this study provides some insight into the genetics underlying the evolution of unique adaptive traits of *M. ovinus* and a new resource for comparative genomic and genetic explorations of the parasites in the family Hippoboscidae.

## Materials and methods

### Sample collection, sequencing, and quality control

A total of 15 adult female specimens of *M. ovinus* were collected from a sheep farm located at Zhidoi County, Yushu Tibetan Autonomous Prefecture, Qinghai province, China (33°37’N, 95°58’E). The species was identified by morphological approaches (Figure S1) and 18S rRNA gene sequence analysis according to previous studies [3]. The specimens were washed extensively in physiological saline and every 5 specimens were pooled with their gut contents removed. The remaining specimens were deposited at the Institute of Zoology, Chinese Academy of Sciences (IZCAS).

Genomic DNA/RNA extraction, library preparation and sequencing were conducted by conducted by the company Berry Genomics (Beijing, China). Total genomic DNA/RNA extraction from pooled keds was conducted according to the instruction of Blood & Cell Culture DNA Kit (Qiagen, USA) and Trizol reagent (Invitrogen, USA), respectively. For long-read sequencing, high quality gDNA (≥ 10 μg, ≥ 100 ng/μL) was prepared for a 20-kb insert SMRTbell library construction using SMRTbell® Express Template Prep Kit 2.0 (Pacific Biosciences, USA) from the pooled DNA isolates and sequenced on the PacBio Sequel platform employing P6-C4 chemistry. For short-read and RNA sequencing, paired-end libraries were constructed with an insert size of 400 bp and sequenced on the Illumina HiSeq X Ten platform (150-nt paired-end reads).

We use clumpify.sh (BBTools suite v37.93, Bushnell) to compressed raw Illumina short reads into clumps and removed duplicates. Quality control was performed with bbduk.sh (BBTools): both sides were trimmed to Q20 based on Phred scores, reads shorter than 15 bp or with more than 5 Ns were discarded, poly-A or poly-T tails of at least 10 bp were trimmed, and overlapping paired reads were corrected.

### Genome size estimation

We employed the strategy of short-read k-mer distributions to estimate the genome size using short sequencing data. The histogram of k-mer frequencies was computed with 17-mers using jellyfish [40]. The genome size was then estimated using GenomeScope v2.0 [41].

### Genome, mitochondrion and transcriptome assembly

We performed de novo genome assembly with long reads using Canu v2.1.1 [42] and wtdbg2 v2.5 [43]. Both assemblies were first polished by Flye v2.4.2 (–polish_target) [44] on raw Pacbio sequences. To improve assembly contiguity, the two assemblies were merged into one assembly after two rounds of quickmerge v0.3 of USAGE 2 [45], and further polished with Illumina short reads using Pilon v1.22 [46]. Subsequently, we filtered possible contaminants by HS-BLASTN [47] and VecScreen against the NCBI and UniVec database, respectively.

We assembled and annotated the mitochondrial genome of *M. ovinus* based on Illumina short reads using MitoZ v2.3 [48] and MITOS webserver [49]. Transcriptome assembly was performed under a genome-guied method, RNA-seq reads to our assembled genome using HISAT2 v2.1.0 [50] and then assembled with StringTie v1.3.4 [51]. Redundant isoforms were removed with Redundans v0.13c [52] under default parameters. Finally, Benchmarking Universal Single-Copy Orthologs (BUSCO) [53] was used to evaluate the completeness of all assemblies.

### Gene prediction and functional annotation

A custom library combining a de novo species-specific repeat library with RepBase-20181026 and Dfam 3.0 databases was generated by RepeatModeler v1.0.11 [54, 55]. Repetitive sequences were predicted by RepeatMasker v4.0.9 together with the custom library [56]. Gene prediction was conducted with the MAKER v2.31.10 pipeline [57] by integrating ab initio predictions, RNA-seq evidence, and protein homology-based searches. For ab initio annotation, Augustus v3.3 [58] and GeneMark-ET v4.33 [59] were simultaneously performed to predict gene coding regions. Two predictors were trained using BRAKER v2.1.0 [60] with RNA-seq data, and previously genome-guided assembled transcripts were used as transcriptome-based evidence. Protein sequences of *Drosophila melanogaster*, *Glossina fuscipes* and *Glossina morsitans* were downloaded as protein homology-based evidence.

Homology-based gene functions were assigned using Diamond v0.9.18 [61] and the UniProtKB (SwissProt + TrEMBL). Protein domains, Gene Ontology (GO) and pathway annotations, were searched with InterProScan 5.34–73.0 [62] against the Pfam [63], PANTHER [64], Gene3D [65], Superfamily [66], and CDD [67] databases. For non-coding genes, ribosomal RNA (rRNA), microRNA (miRNA), and small nuclear RNA (snRNA) genes were predicted by Infernal v1.1.2 [68] against the Rfam v14.0 [69] database. Transfer RNAs were further refined using tRNAscan-SE v2.0 [70].

### Gene family analysis and evolution

We identified gene families using 10 public genome protein sequences of insect species, including *Aedes aegypti*, *Anopheles gambiae*, *Bactrocera oleae*, *Ceratitis capitate*, *Drosophila melanogaster*,*Glossina fuscipes*,*Lucilia cuprina*, *Musca domestica*, *Sarcophaga bullata*, and *Stomoxys calcitrans*. *Aedes aegypti* and *Anopheles gambiae* were selected as the out-group. OrthoFinder v2.2.7 [71] was used to infer orthogroups with Diamond [61] as the sequence aligner. Gene family evolution (gain and loss) was analyzed using CAFE v4.2 [72] with the lambda parameter used to calculate birth and death rates. The ultrametric tree generated from OrthoFinder was transformed using r8s [73] and time-calibrated by the divergence time (35 mya) of the most recent common ancestor between *Musca domestica* and *Stomoxys calcitrans* from the TimeTree database [74].

### Phylogenetic analysis

The identified sequences of *mgp* genes, PGRP genes, sensory receptors gene, and vision-associated rhodopsin genes were combined with the corresponding homologous genes of *Drosophila melanogaster*, *Musca domestica*, *Glossina fuscipes* and *Glossina morsitans* and used as template sequences to homologous blast with the *M. ovinus* genome database (with e-value as 1e-5) to determine the annotation results of gene families [75]. The protein sequences of the target genes were aligned using MAFFT software [76]. The phylogenetic tree reconstructions were made using maximum likelihood (ML) based on IQ-TREE v2 [77] with 1,000 ultrafast bootstraps. Dendrograms were edited using Interactive Tree of Life (iTOL) v5 [78].

### Reverse transcription quantitative PCR (RT-qPCR)

Total RNAs were isolated from female and male keds with Trizol reagent (Invitrogen, USA). cDNA templates were synthesized from 1 μg RNA using the GoScript Reverse Transcription System (Promega, USA). Specific primers for *mgp* genes and Tubulin were designed with the identified gene sequences by genome analysis (Table S8). The relative expression levels were quantified using the SYBR Green qPCR kit (Cwbio, CHINA) and Applied Biosystems 7500 Fast Real-Time PCR system (Thermo, USA). Expression of target genes were normalized to the reference endogenous Tubulin and calculated by using the 2^−ΔΔCt^ method. Statistical analysis was analyzed by Student’s *t*-test using GraphPad Prism 7 (GraphPad).

## Supplementary Information

**Additional file 1:** **Figure S1. **Morphological characteristics of *M. ovinus*. A: Dorsal view of the female; B: Ventral view of the female; C: Dorsal view of the male; D: Ventral view of the male.** Figure S2.** GenomeScope results for *M. ovinus*. A: untransformed linear plot; B: untransformed log plot; C: transformed linear plot; D: transformed log plot. **Figure S3.** A comparative representation of orthologous and paralogous genes aligned with other ten insect genomes.** Figure S4.** Rapidly evolving gene families (gene family expansion).** Figure S5.** Phylogenetic analysis of yolk protein genes from *M. ovinus *and ten other species.

**Additional file 2:** **Table S1. **Summary of each assembly at each step for *M. ovinus*. **Table S2.** GenomeScope genome size estimates for *M. ovinus* with different combinations of k-mer length and maximum k-mer coverage. **Table S3.** Repeat annotation in *M. ovinus*. **Table S4.** Summary statistics for non-coding RNAs in *M. ovinus*. **Table S5.** Summary statistics for gene family inference and gene counts for each family and each species. **Table S6.** Gene family evolution reported from CAFE. **Table S7.** Rapidly evolving gene families in *M. ovinus*. **Table S8.** Primers used in the present study.

## Data Availability

Raw sequencing data are deposited in the NCBI Sequence Read Archive under the accessions SRR17267914-SRR17267916. The genome and transcriptome assemblies are available at GenBank with the accession number SRX13445939, SRX13445940, respectively, corresponding to the BioProject PRJNA789604 and the BioSample SAMN24146076. All supplementary figures and tables are provided as Additional File.

## Abbreviations

CSPs: Chemosensory proteins
GRs: Gustatory receptors
OBPs: Odorant Binding Proteins
ORs: Odorant Receptors
IRs: Ionotropic Receptors,
SNMPs: Sensory Neuron Membrane Proteins
