# Supplementary material for: The genome of sheep ked (Melophagus ovinus) reveals potential mechanisms underlying reproduction and narrower ecological niches
Source: BMC Genomics. 2023 Jan 30;24:54. doi: 10.1186/s12864-023-09155-1 (PMC9887928; doi:10.1186/s12864-023-09155-1)
Supplement: Supplementary file 1 — Additional file 1: Figure S1. Morphological characteristics of M. ovinus. A: Dorsal view of the female; B: Ventral view of the female; C: Dorsal view of the male; D: Ventral view of the male. Figure S2. GenomeScope results for M. ovinus. A: untransformed linear plot; B: untransformed log plot; C: transformed linear plot; D: transformed log plot. Figure S3. A comparative representation of orthologous and paralogous genes aligned with other ten insect genomes. Figure S4. Rapidly evolving gene families (gene family expansion). Figure S5. Phylogenetic analysis of yolk protein genes from M. ovinus and ten other species. [file 12864_2023_9155_MOESM1_ESM.docx]

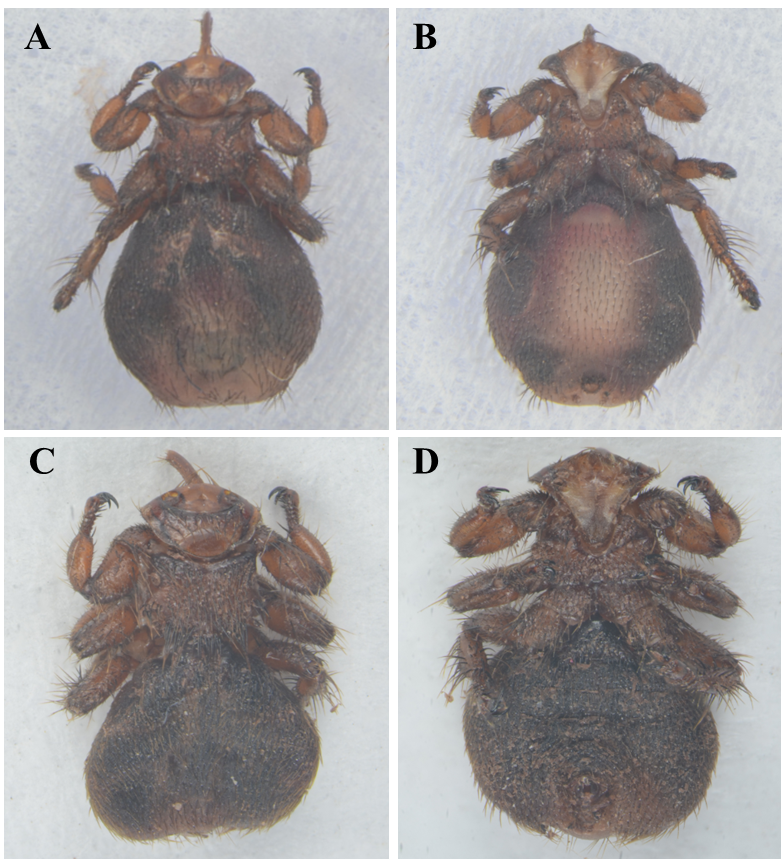


**Figure S1**. Morphological characteristics of *M. ovinus*. A: Dorsal view of the female; B: Ventral view of the female; C: Dorsal view of the male; D: Ventral view of the male.


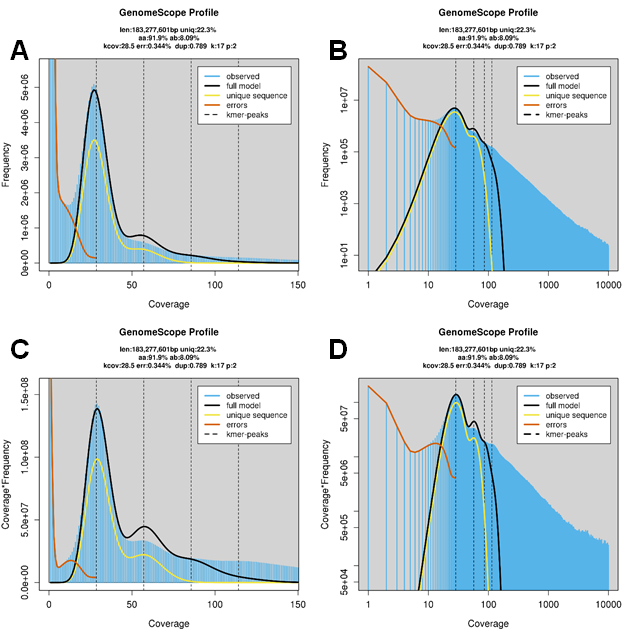


**Figure S2**. GenomeScope results for *M. ovinus*. A: untransformed linear plot; B: untransformed log plot; C: transformed linear plot; D: transformed log plot.


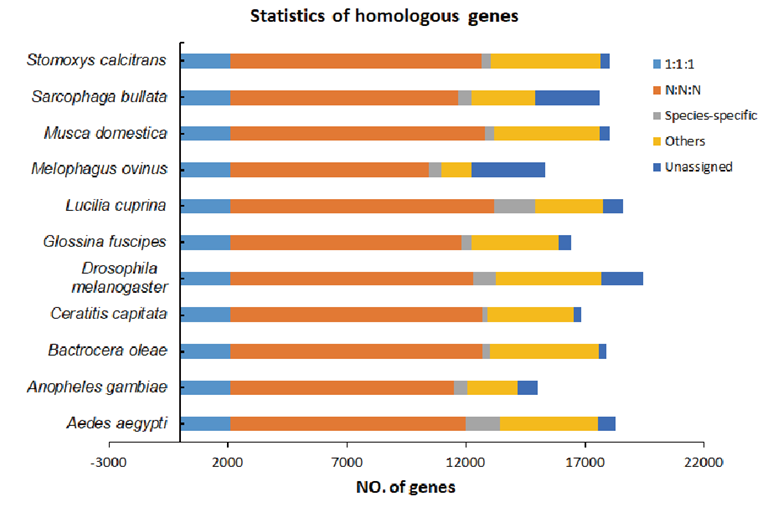


**Figure S3**. A comparative representation of orthologous and paralogous genes aligned with other ten insect genomes.


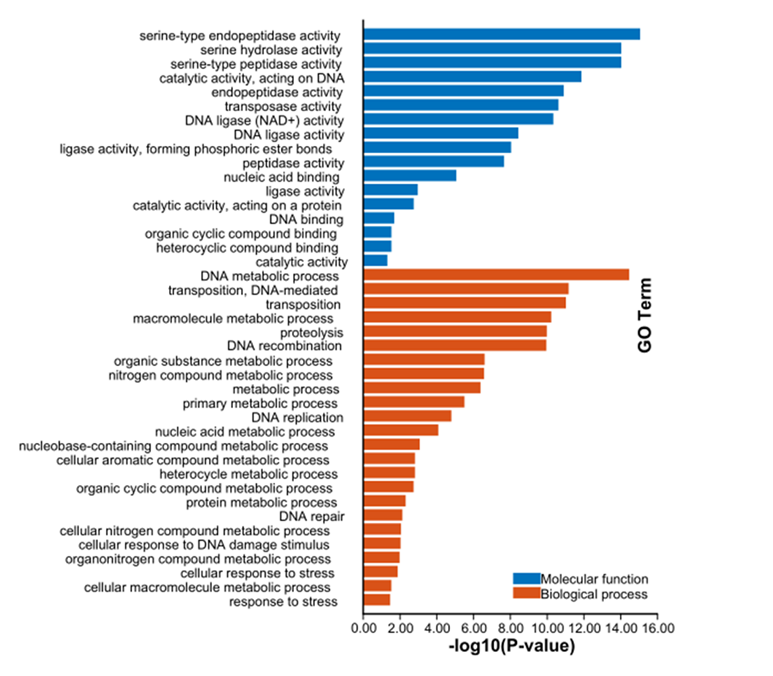


**Figure S4**. Rapidly evolving gene families (gene family expansion).


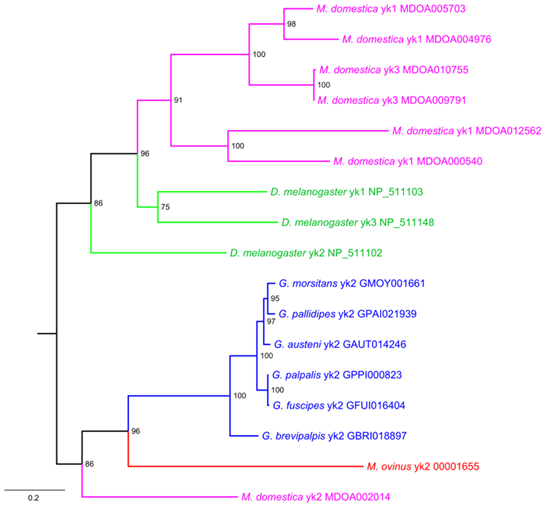


**Figure S5**. Phylogenetic analysis of yolk protein genes from *M. ovinus* and ten other species.
